# Pharmacogenomics knowledge and implementation readiness among community pharmacists in Jordan: A national cross-sectional study

**DOI:** 10.1371/journal.pone.0349439

**Published:** 2026-05-19

**Authors:** Khalid Awad Al-Kubaisi, Derar H. Abdel-Qader, Nadia Al Mazrouei, Rana Ibrahim, Ahmed Alhusban

**Affiliations:** 1 College of Pharmacy, Department of Pharmacy Practice and Pharmacotherapeutics, University of Sharjah, Sharjah, United Arab Emirates; 2 Faculty of Pharmacy and Medical Science, University of Petra, Amman, Jordan; 3 College of Pharmacy, University of Sharjah, Sharjah, United Arab Emirates; 4 Department of Pharmacy Practice and Pharmacotherapeutics, College of Pharmacy, University of Sharjah, Sharjah, United Arab Emirates; University of Petra (UOP), JORDAN

## Abstract

Pharmacogenomics (PGx) offers a powerful strategy to improve medication safety and efficacy, yet its integration into community pharmacy practice remains limited. While an “attitude-knowledge gap” is known to exist globally, limited national data exist concerning PGx readiness among community pharmacists in Jordan. This study aimed to (i) quantify Clinical Pharmacogenetics Implementation Consortium (CPIC)-aligned PGx knowledge and characterize attitudes; (ii) assess implementation readiness across organizational, leadership, and structural dimensions; (iii) map perceived barriers; and (iv) identify independent predictors of knowledge, attitudes, and readiness. A national, cross-sectional survey was conducted using a proportionate stratified random sampling method. A content-validated and reliability-tested electronic questionnaire was distributed to community pharmacists across Jordan’s three administrative regions. The instrument assessed demographics, CPIC-aligned knowledge, attitudes and perceived barriers on five-point Likert scales, and theory-informed implementation readiness. Data were analyzed using descriptive statistics, bivariate tests, and multiple linear regression. A total of 347 community pharmacists completed the survey. A vast majority (83.0%) reported no prior formal PGx training. Knowledge of practical PGx was low, with a mean score of 5.54 ± 2.12 out of 10. Attitudes were neutral (mean = 3.49 ± 0.26), while perceived barriers were rated as significant (mean severity = 3.45 ± 0.30), with privacy concerns being the highest-rated challenge (mean = 3.65 ± 0.78). Total implementation readiness was moderate (mean = 3.34 ± 0.25), with organizational readiness (mean = 3.39) rated higher than structural readiness (mean = 3.21) or leadership support (mean = 3.27). Bivariate analyses revealed few significant associations, and crucially, multiple linear regression models showed that no demographic or practice characteristics within the variables tested were significant independent predictors of knowledge or readiness. Jordanian community pharmacists demonstrated a significant gap between their positive attitudes towards PGx and the practical knowledge and systemic support required for clinical implementation. The findings revealed that the identified deficits in knowledge and readiness were widespread across all surveyed subgroups, not concentrated in specific demographic or professional subgroups. Translating the existing positive sentiment into routine clinical practice urgently requires a nationwide, standardized educational strategy coupled with the development of systemic enablers, including reimbursement pathways, integrated clinical workflows, and explicit leadership support.

## Introduction

Interindividual variability in drug response is an important factor contributing to preventable adverse drug reactions and suboptimal therapeutic outcomes. Pharmacogenomics (PGx), the tailoring of pharmacotherapy based on a patient’s genotype, represents an important approach to achieve this goal. Recent evidence synthesis studies demonstrated evidence for the potential of PGx-guided pharmacotherapy to decrease adverse drug reactions, as well as enhance therapeutic outcomes, even under clinical settings [1]. Moreover, to translate this evidence base into successful clinical applications, the Clinical Pharmacogenetics Implementation Consortium [CPIC), has established evidence-grade, peer-reviewed pharmacotherapy guidelines, which define the evidence-based recommendations clinicians should follow to personalize pharmacotherapy decisions according to their genotype [2].

As medication specialists, pharmacists, especially those engaged in a community-based delivery model, are seen as having the prime potential to incorporate PGx-based care, including education, interpretation, and medication adjustment, as a service interaction involving prescribers. Various professional organizations, including the American Society of Health-System Pharmacists (ASHP), have issued position statements outlining pharmacists’ central responsibility in the ethical and evidence-based utilization of clinical pharmacogenomics, respectively [3,4]. Still, the integration of PGx into various community-based pharmacy services continues to remain underutilized, even as a viable evidence-based care approach exists in relation to various pharmacology applications.

An emerging international literature demonstrates a consistent finding: pharmacists and pharmacy students tend to express a positive attitude toward PGx, but express a lack of know-how or confidence to seriously bring this technology into actual clinical utility. The attitude-knowledge gap can often be exacerbated by systemic barriers related to work flow, reimbursement, and information technology, among other areas [5]. The findings among the international literature described are particularly represented by examples related to the prevalence among the Middle East region countries, including Kuwait, the UAE, and Qatar [6–8]. The difficulty has been encapsulated by a recognized regional assertion that a two-tiered strategy focusing both on standardized education as well as well-structured implementation approaches will be required to ameliorate this attitude-knowledge dissociation between actual preference and actual viability as described by Khattab et al. [9] among others. Current literature consistently shows a positive attitude toward PGx across the Middle East; however, these studies often overlook the systemic barriers such as reimbursement and IT infrastructure that are critical for actual practice change. Moreover, while previous regional studies have assessed PGx knowledge, there is a lack of evidence regarding implementation readiness—specifically organizational and structural capacity—within the Jordanian community pharmacy context.

To bridge this gap from individual sentiment to systemic practice, a comprehensive national benchmark is required. This study aimed to address this gap by (i) quantifying CPIC-aligned PGx knowledge and characterizing attitudes among Jordanian community pharmacists; (ii) assessing implementation readiness across organizational, leadership, structural, and privacy dimensions; (iii) identify the primary systemic barriers to service adoption; and (iv) determine whether demographic factors or practice settings serve as independent predictors of readiness. By providing a comprehensive national benchmark, this research sought to inform the development of targeted educational and systems-level strategies to facilitate the adoption of pharmacogenomics in Jordanian community pharmacy.

Despite these regional insights, a critical research gap persists in the Jordanian context. Existing studies have largely focused on the individual pharmacist’s perception, leaving the organizational and structural dimensions of implementation readiness—such as leadership support, IT infrastructure, and privacy framework—entirely unexplored. Without understanding whether the community pharmacy environment is systemically prepared to host PGx services, educational interventions alone may fail to translate into practice. This study addresses this void by providing the first national assessment that integrates individual knowledge and attitudes with theory-informed implementation science constructs to determine the true readiness of the Jordanian pharmacy sector.

## Materials and methods

### Study design and setting

A national, cross-sectional survey was conducted to assess the knowledge, attitudes, perceived barriers, and implementation readiness for PGx services among community pharmacists in Jordan. The study was conducted between August and October, 2025, and data were gathered among pharmacists distributed across all three primary administrative areas in Jordan.

### Study population and sampling

The population included all licensed pharmacists actively engaged in their professions within community pharmacies within the Hashemite Kingdom of Jordan. The stratified random proportionate sampling method was applied to ensure that a nationally representative sample was produced, taking into account the geographical distribution of community pharmacies. The sampling frame was derived from the official registry maintained by the Jordan Pharmacists Association (JPA), with the study population consisting of 3,576 licensed community pharmacies operating within Jordan during the conduct of this research. The population was subdivided into three groups that comprised non-overlapping strata defined by such geographical distribution. These groups are the Central Region with 2,519 (70.4%), the North Region with 855 (23.9%), and the South Region with 202 (5.6%).

Participants were required to meet all of the following inclusion criteria to be eligible for the study: holding a valid license to practice pharmacy in Jordan, currently and actively practicing in a community pharmacy setting and providing electronic informed consent to participate. Pharmacists practicing in non-community settings (e.g., hospitals, industry, academia), individuals practicing as intern or trainee pharmacists and pharmacists who declined to provide informed consent were excluded from the study.

The sample size was calculated a priori to ensure that the research had sufficient statistical power to enable it to answer with 95% confidence within 5% margin error. The initial sample was set to 385 using Cochran’s formula (n = [Z² * P(1-P)]/ E²), with an initial conservative estimation that 50% would respond to the question to allow maximum variability within the sample. Since this was above 5% of the population proportion in the finite population study group, the study made use of the Finite Population Correction to improve efficiency [10], so that the resultant sample to be sought was 347. The sample sought was proportionately split among the strata to get 244 in the Central Region, 83 in the North Region, and 20 in the South Region. A post-hoc power analysis for the multiple linear regression models (with 11 predictors) confirmed that a sample size of 347 provided over 95% power to detect a medium effect size (f2 = 0.15) at a significance level of α = 0.05.

The process of selecting the study subjects entailed randomly sampling the requisite numbers of pharmacies from the lists that had been compiled for each of the strata utilizing a computer-based random number generator. To ensure consistency and minimize selection bias, a subsequent invitation was extended specifically to the pharmacist-in-charge or a designated senior staff pharmacist from each of these selected pharmacies. This approach ensured that the respondent possessed a comprehensive overview of the pharmacy’s clinical operations and leadership support structures.

### Research instrument

To facilitate data collection, an electronic questionnaire (e-questionnaire) was designed to be new self-administered by study subjects. The e-questionnaire was developed as a hybrid tool consisting of adapted items from internationally validated surveys and original items tailored to the Jordanian context from previous reviews [1,5,8,9,11–22]. Items were modified to ensure relevance to local practice. For example, the privacy items were updated to specifically reference compliance with the Jordanian Personal Data Protection Law (No. 24/ 2023 PDPL). Furthermore, all technical drug-gene pair questions were cross-referenced with the latest CPIC guidelines [2] to ensure clinical accuracy.

The final instrument was segmented to include six different sections. The first section was complete with a participant information sheet and an entire consent section. The second substantive section requested demographic and practice data.

The third section was the pharmacogenomic knowledge assessment section, which was comprised of a ten multiple-choice question section rated with a potential score that ranged from 0 to 10 where each question anchored to the guidelines established by the CPIC [11]. The section was scored by allocating one point to every correct answer with zero points allotted to every incorrect answer, with ‘I don’t know’ answers also attracting zero points. For descriptive purposes, scores were categorized as ‘low’ (<6/10) or ‘high’ (≥ 6/10) This cutoff was selected to ensure consistency and facilitate direct comparison with previous regional studies where similar scoring systems were established [8].The fourth section aimed to determine the attitudes and perceptions of pharmacists by means of nine declarative statements using a five-point Likert scaling format with ‘1’ corresponding to ‘Strongly Disagree’ and ‘5’ to ‘Strongly Agree’ that are all formulated in a positive form. In data analysis, the average of each respondent’s responses to all nine items provides an accurate continuous variable with values ranging from 1 to 5 to reflect the magnitude that depicts greater positivity among the subjects. A composite mean attitude score was derived by averaging responses. While Likert scales are inherently ordinal, treating the averaged composite score as a quasi-continuous variable is an established practice in health services research to allow for robust parametric analysis [23].

The fifth section aimed to identify barriers to the implementation process and evaluate the level of their severity using a five-point Likert scaling format with ‘1’ indicating ‘Not a Barrier’ and ‘5’ indicating ‘A Critical Barrier.’ The processed data were averaged to rank barriers according to their relative degree by calculating the average level of each barrier to identify the most severe barriers.

The last part of the questionnaire included the measurement of the readiness to implement, addressed by a set of targeted items consisting of ten statements rated using a five-point Likert scale from ‘1’ (Strongly Disagree) to ‘5’ (Strongly Agree). These items were theory-informed, adapting the core constructs of established implementation science frameworks. Specifically, single items were formulated to represent organizational commitment and efficacy (adapted from the Organizational Readiness for Implementing Change scale, ORIC) [20], leadership support (adapted from the Implementation Leadership Scale, ILS) [21], and concerns regarding patient privacy (adapted from the Internet Users’ Information Privacy Concerns Scale, IUIPC) [22]. For analytical purposes, the items were grouped into several sub-constructs. A composite Organizational Readiness score was calculated by averaging the two items on collective commitment and capability. Similarly, Structural Readiness and Personal Readiness (self-efficacy and intention) scores were calculated by averaging their respective items. The items measuring leadership support and privacy concerns were analyzed descriptively as individual indicators. These items were theory-informed, adapting core constructs from the ORIC, ILS, and IUIPC frameworks. However, it should be noted that these represent high-impact proxy statements rather than full validated scales, and were selected to minimize respondent fatigue while maintaining conceptual alignment with implementation science.

The full instrument is provided in Supplementary Material S1.

To establish content validity, the e-questionnaire was peer-reviewed to ensure that it was valid in terms of content with an expert group comprising 2 academicians with expertise in pharmacy practice, an expert geneticist with expertise in genetic medicine, and 2 senior community pharmacists. They rated each item’s relevance on a four-point scale (1 = Not Relevant to 4 = Very Relevant). The Item-Level Content Validity Index (I-CVI) was calculated as the proportion of experts giving a rating of 3 or 4 and ranged from 0.80 to 1.0. The overall Scale-Level Content Validity Index (S-CVI/AVE) was calculated as the average of the I-CVI values, resulting in an index of 0.92, indicating excellent consensus.

The reliability of the multi-item scales was assessed through internal consistency and test-retest reliability. Internal consistency, measured by Cronbach’s alpha, was calculated for the following constructs: Attitudes (α = 0.82), Perceived Barriers (α = 0.78), and Total Implementation Readiness (α = 0.85), all of which exceeded the acceptable threshold of 0.70. Due to the use of theory-informed proxy items rather than full scales, exploratory factor analysis (EFA) was not performed; instead, construct validity was supported by the adaptation of items from previously validated primary studies [8,11,20].

Furthermore, the e-questionnaire was pilot-tested with 20 community pharmacists to ensure that it was clear and valid. The test-retest reliability was evaluated during the pilot study (n = 20) by administering the questionnaire twice with a two-week interval. The Intraclass Correlation Coefficient (ICC) for the composite readiness score was 0.88 (95% CI: 0.81–0.93), indicating excellent stability over time.

### Data collection procedure

Pursuant to the approval given by the Institutional Review Board (IRB) at the University of Petra, an endorsement was requested from the JPA to facilitate gaining access to the sampling frame to support the conduct of this study.

The data acquisition procedure took place completely electronically over a period of 12 weeks. A master list with a set of randomly selected community pharmacies, proportionally stratified by region, was produced as described before. The invitation to participate followed as described, being sent to one pharmacist per selected community pharmacy via email or a direct WhatsApp® message. The initial invitation included a brief introductory part, describing the significance and purpose of the study, a commitment to anonymity and confidentiality, as well as a unique, non-transferable link to the e-questionnaire hosted on an internet-based secure web platform.

A systematic follow-up approach was adopted to achieve the highest response rate. Automated reminder emails/messages were sent to all non-responders exactly two weeks and four weeks following the initial invitation. The reminders politely repeated the significance of the study and included the link to the survey once again. The survey link was programmed to prevent duplicate submissions by the same respondent. The period allowed for data collection remained open until the targeted sample size was reached for each of the three geographical strata, so as to preserve the proportionate integrity of the final sample. Once the survey was completed, the data were secured by downloading, anonymized by stripping any possible indirect identifiers, and processed for statistical analysis.

### Ethical considerations

This study was conducted in accordance with the principles of the Declaration of Helsinki. Ethical approval was obtained from the University of Petra IRB (S/17/8/2025). All participants were provided with a detailed information sheet and were required to provide electronic informed consent before proceeding with the survey. Participation was anonymous, and all data were kept confidential and stored on a secure, password-protected server.

### Statistical analysis

The data were analyzed with the SPSS Statistics Version 28 software program produced by IBM Corporation, Armonk, NY. The interpretation criteria for the five-point Likert scales are as follow: values ranging from 1.00 to 2.49 are interpreted as ‘negative/low’, values ranging from 2.50 to 3.49 are interpreted as ‘neutral/ moderate’, and values ranging from 3.50 to 5.00 are interpreted as ‘positive/high’ respectively [23,24].

The data were described using descriptive statistics, which included frequencies, percentages, means, and standard deviations (SD). The choice between parametric and non-parametric tests was determined after assessing the data for normality. For normally distributed data, parametric tests such as the independent t-test (for two groups) and Analysis of Variance (ANOVA) (for three or more groups) were used to compare mean scores. Data normality was assessed using the Shapiro-Wilk test. In cases where the assumption of normality was not met, the non-parametric alternatives, the Mann-Whitney U test and the Kruskal-Wallis test, were employed, respectively. This approach ensures the appropriate statistical analysis based on the distribution of the data. The significance level was set at p-value < 0.05, for all tests.

Lastly, three multiple regression models were developed to examine the independent predicting factors for each primary outcome, namely knowledge score, total implementation readiness, and attitude score. Multiple linear regression was chosen because the primary outcomes of interest, knowledge, readiness, and attitude scores, were all measured as continuous variables. The potential independent predicting factors included all the demographic and practice-based variables, which were included as potential predictive factors in each model. For the regression models, assumptions of linearity, homoscedasticity, and normality of residuals were verified through the inspection of P-P plots and scatterplots of standardized residuals.

Before running the regression, multicollinearity was assessed; all actual VIF values were found to be low (ranging from 1.12 to 1.84), well below the established threshold of 5, indicating no significant threat to the regression results. The standardized beta coefficients (β) and their respective p-values included in the results allowed verification whether each independent predictive factor significantly predicted the outcome factor.

## Results

A total of 347 community pharmacists from across Jordan completed the survey, meeting the target sample size.

### Participant demographic and practice characteristics

A total of 520 electronic invitations were distributed to community pharmacists across the three administrative regions of Jordan. From these, 347 pharmacists completed the survey, resulting in an overall response rate of 66.7%.

The demographic and professional parameters of participants are given in Table 1. The participants were almost equally divided between the two genders, with 178 female participants (51.3%) and 169 male participants (48.7%). The geographic distribution of participants paralleled the proportionate stratified study plan, with 244 participants (70.3%) being practitioners from the Central Region, 83 participants (23.9%) from the North, and 20 participants (5.8%) from the South Regions, respectively.

**Table 1:** Participant demographic and practice characteristics (N = 347).

Characteristic	Category	Count (n)	Percent (%)
**Gender**	Female	178	51.3
	Male	169	48.7
**Region**	Central	244	70.3
	North	83	23.9
	South	20	5.8
**Highest Degree**	BSc in Pharmacy	258	74.4
	PharmD	61	17.6
	MSc or higher	28	8.1
**Years of Practice**	0-5 years	119	34.3
	6-10 years	90	25.9
	11-20 years	102	29.4
	>20 years	36	10.4
**Pharmacy Type**	Independent	250	72.1
	Chain	97	27.9
**Prior PGx Training**	No	288	83.0
	Yes	59	17.0

The majority of the participants (258, 74.4%), possessed a Bachelor of Science degree in Pharmacy as their highest academic qualification, 61 participants (17.6%) possessed a Doctor of Pharmacy degree, and 28 participants (8.1%) possessed a Master’s degree or higher. A diverse number of participants possessed varying levels of professional experience, with the highest number (119, 34.3%), having worked between 0–5 years as pharmacists. The majority (250) worked in private or independently owned pharmacies, which constituted 72.1% of the total number of participants. One of the important findings was that a large number of participants (288, 83.0%), confirmed that they had received no former training in pharmacogenomics.

### Pharmacogenomic knowledge assessment

The pharmacogenomic level of understanding among the participants was measured by a questionnaire consisting of ten multiple-choice questions. The results indicated moderate knowledge levels, as measured by the criteria predetermined for this study. Having a mean score of 5.54 ± 2.12, as measured by a classification system whereby a score of less than 6 out of 10 represented ‘low’, indicated that this was indeed the case, reflecting a combined correct response rate of 55.4%. This suggests a foundational understanding of PGx principles that nonetheless requires further clinical refinement for practical implementation.

The percentage of subjects answering each question correctly is presented in Table 2. The response rates remained reasonably consistent, varying between a low of 51.0% and a high of 60.5%. The question with the highest response rate of 60.5% related to the primary purpose of pharmacogenetic testing (K6), whereas the question regarding the most appropriate concept of counseling a PGx test result (K10) was responded to correctly by the lowest percentage of subjects, 51.0%. The question related to each of the well-known interactions between a drug and a gene, clopidogrel (K3, 58.8%), codeine (K2, 56.8%), and warfarin (K4, 52.2%), were all responded to correctly by just over 50% of the subjects.

**Table 2:** Pharmacogenomic knowledge assessment results (N = 347).

Question Abbreviation	Correct Count (n)	Correct Percent (%)
**K1:** Definition of PGx	185	53.3
**K2:** Codeine & CYP2D6 Poor Metabolizer	197	56.8
**K3:** Clopidogrel & CYP2C19 gene	204	58.8
**K4:** Warfarin key genes	181	52.2
**K5:** Citalopram & CYP2C19 Ultra-Rapid Metabolizer	179	51.6
**K6:** Primary use of PGx testing	210	60.5
**K7:** Allopurinol & HLA-B*58:01	206	59.4
**K8:** Most reliable PGx resource (CPIC)	192	55.3
**K9:** Azathioprine & TPMT Poor Metabolizer	190	54.8
**K10:** Appropriate counseling concept for a PGx result	177	51.0

### Attitudes, perceived barriers, and implementation readiness

The rest of the scale measured the attitude of pharmacists towards PGx, the perceived barriers to the implementation of PGx, and their readiness to offer the service as a whole. Composite scores were obtained for each of the mentioned scales, each measured by a five-point scale. A summary of the results is detailed in Table 3.

**Table 3:** Composite scores for attitudes, barriers, and implementation readiness (N = 347).

Composite Score	N	Mean	SD	95% CI	Min	Median	Max
**Attitude**	347	3.49	0.26	**[3.46, 3.52]**	2.89	3.56	4.33
**Barriers (Severity)**	347	3.45	0.3	**[3.42, 3.48]**	2.86	3.43	4.43
**Total Implementation Readiness**	347	3.34	0.25	**[3.31, 3.37]**	2.3	3.3	4.1
Organizational Readiness	347	3.39	0.56	**[3.33, 3.45]**	2	3.5	5
Leadership Support	347	3.27	0.81	**[3.18, 3.36]**	1	3	5
Structural & Practical Readiness	347	3.21	0.57	**[3.15, 3.27]**	1.5	3.5	4.5
Privacy Concerns	347	3.65	0.78	**[3.57, 3.73]**	2	4	5
Personal Readiness and Intention	347	3.31	0.4	**[3.27, 3.35]**	2.25	3.25	4.25

The overall attitude presented by community pharmacists towards the professional utility and value for clinical applications of pharmacogenomics remained relatively neutral. The overall attitude score was 3.49, with a standard deviation of 0.26, as measured by the five-point Likert scale. The attitude remained relatively centralized around a positively stated position, with a median position of 3.56, reflective of an acceptance, even minded rather than overly enthusiastic, as indicated by the relatively low standard deviation. The overall attitude did remain centralized between a low score position of 2.89 and a high score position of 4.33.

The participants rated the scale of barriers to providing PGx services as being relatively important. The overall mean score for barriers’ gravity was 3.45 (SD = 0.30). It lies between ‘Moderate Barrier’ (3) and ‘Major Barrier’ (4). The slight difference was seen when comparing with Privacy Concerns, which had a mean score 3.65 (SD = 0.78). The highest-ranked individual item, as mentioned, ratified that any concerns regarding patients’ confidentiality, as well as the requirements of the PDPL, were perceived as the most serious barriers by the pharmacists, indeed being the most important ones. The medium score was 3.43, once again affirming that, relatively, pharmacists regard the actual barriers as serious enough to prevent successful implementation.

A relative ranking of the seven perceived barriers by mean severity identified ‘Privacy Concerns’ (M = 3.65) as the primary challenge, followed by ‘Lack of reimbursement’ (M = 3.48) and ‘High out-of-pocket costs’ (M = 3.45). This hierarchical distribution confirms that systemic and economic factors are perceived as more significant hindrances than individual reluctance.

Multiple dimensions of readiness were assessed, and results indicated a complex, moderately ready condition. The score related to Total Implementation Readiness was 3.34, with a standard deviation of 0.25, signifying a cautiously optimistic attitude rather than a readiness posture.

When looking at the sub-constructs, the highest mean score was for Organizational Readiness, indicating commitment and organizational capability, with a mean score of 3.39 (SD = 0.56), and a median of 3.5, implying a moderately positive attitude that organizations could be open to new service developments. The lowest two were, as hypothesized, the Structural & Practical Readiness score, with a mean score of 3.21 (SD = 0.57), and the Leadership Support, with a mean score of 3.27 (SD = 0.81), reconfirming that the lack of actual resource availability, including private counseling areas and suitable IT, as well as apparent leadership support, were central findings as barriers to development. The sub-construct, Personal Readiness and Intention, reflecting self-efficacy and future intention, achieved a mean score of 3.31 (SD = 0.40). The central tendency, well clustered around the median of 3.25, indicated a moderately optimistic attitude among pharmacists regarding their capabilities and ambitions to make PGx a future reality.

### Bivariate analysis of factors associated with outcome scores

A series of bivariate tests were performed to identify the demographic/practice related factors that influence the pharmacists’ knowledge, attitude, and total readiness related to PGx. The mean values of each outcome were compared among all the subgroups identified. The results are presented in Table 4.

**Table 4:** Bivariate analysis of factors associated with primary outcome scores.

Characteristic	Category	Knowledge Score (0–10)	Attitude Score (1–5)	Total Implementation Readiness Score (1–5)	Effect Size
**Gender**	Female	5.48 ± 2.15	3.47 ± 0.26	3.33 ± 0.25	d = 0.05
	Male	5.60 ± 2.10	3.50 ± 0.26	3.35 ± 0.25	
	p-value	0.60	0.30	0.68	
**Region**	Central	5.59 ± 2.07	3.49 ± 0.26	3.35 ± 0.24	η² = 0.003
	North	5.57 ± 2.24	3.48 ± 0.27	3.33 ± 0.26	
	South	4.70 ± 2.15	3.46 ± 0.16	3.31 ± 0.23	
	p-value	0.19	0.81	0.62	
**Highest Degree**	BSc in Pharmacy	5.59 ± 2.02	3.48 ± 0.25	3.33 ± 0.24	η² = 0.010
	PharmD	5.57 ± 2.47	3.49 ± 0.30	3.34 ± 0.28	
	MSc or higher	4.93 ± 2.18	3.54 ± 0.27	3.37 ± 0.23	
	p-value	0.29	0.55	0.48	
**Years of Practice**	0-5	5.75 ± 2.16	3.48 ± 0.23	3.35 ± 0.23	η² = 0.006
	6-10	5.33 ± 1.83	3.47 ± 0.25	3.33 ± 0.23	
	11-20	5.55 ± 2.20	3.50 ± 0.29	3.33 ± 0.27	
	>20	5.31 ± 2.42	3.52 ± 0.27	3.31 ± 0.29	
	p-value	0.49	0.73	0.57	
**Pharmacy Type**	Chain	5.55 ± 2.05	3.48 ± 0.26	3.36 ± 0.24	
	Independent	5.53 ± 2.15	3.49 ± 0.26	3.33 ± 0.25	
	p-value	0.95	0.70	0.21	
**Prior PGx Training**	No	5.59 ± 2.13	3.48 ± 0.27	3.33 ± 0.25	d = 0.26
	Yes	5.27 ± 2.08	3.54 ± 0.20	3.38 ± 0.23	for Attitude
	p-value	0.29	0.04*	0.08	

Note: Bold p-value indicates statistical significance (p < 0.05).

Note: Cohen’s d was used for two-group comparisons, and η² was used for comparisons involving three or more groups.

Abbreviation: PGx, pharmacogenomics.

The results showed a marked absence of statistically significant associations between the traditional demographic and practice variables and the primary outcome scores. Based on both the Knowledge Score and the Total Implementation Readiness Score, there were no statistically significant differences as a function of any of the tested variables, including gender, region, highest academic degree, number of years as a practitioner, type of pharmacy, and experience with PGx training, respectively (p > 0.05 each). The absence of statistically significant associations between the selected demographic and practice variables and the primary outcome scores indicated that the community pharmacists’ low knowledge level and moderate implementation readiness are evenly distributed among all members comprising the population. However, it must be acknowledged that this lack of significance could also be influenced by inherent measurement error in self-reported data or the limited sensitivity of the proxy items used to detect subtle differences between specific professional subgroups.

The lone statistically significant result that came out of the analysis was found when examining Pharmacists’ Attitudes. Pharmacists with past formal training in PGx expressed a statistically significantly more favorable attitude score mean (M = 3.54, SD = 0.20) than pharmacists with no past training (M = 3.48, SD = 0.27, p = 0.04). While past training failed to significantly influence either knowledge or overall readiness, this unadjusted analysis reveals that past training was a statistically significant predictor of a strong attitude toward the value and utility of pharmacogenomics.

### Correlation analysis

To explore the relationships between the primary measured constructs, a pearson correlation analysis was performed (Table 5). A statistically significant positive correlation was found between attitudes and total implementation readiness (r = 0.42, p < 0.001), suggesting that more favorable attitudes are associated with higher perceived readiness. However, knowledge scores did not significantly correlate with either attitude (r = 0.08, p = 0.14) or readiness (r = 0.05, p = 0.32).

**Table 5:** Pearson correlation matrix of measured constructs (N = 347).

Construct	1. Knowledge	2. Attitude	3. Barriers	4. Readiness
1	1			
2	0.08	1		
3	−0.12	−0.05	1	
4	0.05	0.42**	−0.21*	1

*p < 0.05, **p < 0.01.

### Multiple linear regression analysis

Three multiple linear regression models were developed to determine the independent predictors for pharmacists’ knowledge, attitude, and overall readiness to implement each PGx service identified. The model tested all of the demographic/practice characteristics surveyed as potential independent variables, regressing each characteristic against all other surveyed characteristics, to determine their independent significance. The results are presented in Table 6.

**Table 6:** Multiple linear regression models predicting knowledge, total implementation readiness, and attitude scores.

Predictor Variable	Model 1: Knowledge Score	Model 2: Attitude Score	Model 3: Total Implementation Readiness Score
	β (p-value)	β (p-value)	β (p-value)
(Constant)			
Prior PGx Training (Ref: No)	−0.05 (0.374)	0.09 (0.094)	0.02 (0.781)
Highest Degree (Ref: BSc)			
PharmD	0.00 (0.996)	0.03 (0.640)	0.01 (0.853)
MSc or higher	−0.08 (0.124)	0.05 (0.345)	−0.02 (0.729)
Years of Practice (Ref: 0–5)			
6–10 years	−0.08 (0.194)	−0.03 (0.672)	0.04 (0.551)
11–20 years	−0.05 (0.446)	0.03 (0.610)	0.01 (0.890)
> 20 years	−0.06 (0.298)	0.04 (0.479)	−0.03 (0.684)
Pharmacy Type (Ref: Independent)	−0.00 (0.958)	−0.02 (0.725)	0.04 (0.512)
Gender (Ref: Male)	−0.03 (0.605)	−0.05 (0.347)	0.03 (0.623)
Knowledge Score (continuous)	–	−0.04 (0.452)	−0.01 (0.815)
Model Fit			
Model F-statistic (df)	1.29 (10, 336)	1.58 (11, 335)	1.35 (11, 335)
Model p-value	0.235	0.106	0.198
Adjusted R²	0.006	0.019	0.008

Note: β represents the standardized beta coefficient. Reference categories for categorical variables are noted in parentheses. No individual predictors were statistically significant (p < 0.05).

The results showed a consistent lack of statistically significant predictors across all three models. The regression model for Knowledge Score was not statistically significant as a whole (F(10, 336) = 1.29, p = 0.235). The model accounted for very little of the variance in knowledge defined by the Knowledge Score ((Adjusted R² = 0.006). The individual predictor variables, highest academic degree or PGx training, were found to be statistically insignificant as a predictor of a pharmacist’s level of knowledge.

Likewise, the model for Total Implementation Readiness (F(11, 335) = 1.35, p = 0.198) and Attitudes (F(11, 335) = 1.58, p = 0.106) were both non-significant as well. Interestingly, the relationship between past PGx training and attitude, which was established as statistically significant in the bivariate analysis (p = 0.04), failed to remain so in the multiple linear regression model (β = 0.09, p = 0.094). It may be seen that, rather than a weak relationship, this could well have been affected by various other confounding factors present in the model.

The consistent absence of significant predictors across all models strongly reinforces the finding that the identified gaps in PGx knowledge and the low level of implementation readiness were universally distributed across the entire population of community pharmacists in Jordan, rather than being concentrated in specific demographic or professional subgroups.

A particularly striking finding of this study was the consistently low explanatory power of the multiple regression models, as indicated by the very small Adjusted R² values for all outcomes (e.g., 0.6% for knowledge). This is not a limitation of the model, but rather a central finding of the research. It revealed that traditional demographic and practice variables, including a pharmacists’ level of academic degree, level of experience, or type of practice, were non-significant indicators of PGx knowledge and readiness among pharmacists today, in this Jordanian scenario.

## Discussion

This national study provided the first comprehensive benchmark of PGx knowledge, attitudes, and implementation readiness among community pharmacists in Jordan. The findings revealed a profession poised at a critical juncture, characterized by a significant gap between positive sentiment and the practical knowledge required for clinical implementation.

A primary finding of this research was the moderate level of PGx knowledge among Jordanian community pharmacists, evidenced by a mean score of 5.54 out of 10. While this score is comparable to regional data from Qatar and Kuwait [8,9], it represents a foundational understanding that requires clinical refinement for practical implementation. This is remarkably consistent with an emerging body of evidence from the broader region where pharmacists express a strong interest in PGx but lack the item-level competence for confident clinical application [5]. Notably, the lack of association between prior training and knowledge scores in the regression model may not signify that education is irrelevant; rather, it likely indicates that existing PGx training in Jordan currently lacks the standardized, clinical depth necessary to yield measurable improvements in practical proficiency.

One of the fascinating results of our research was that the traditional indicators of expertise, including holding a PharmD or having any PGx training, did not prove to be statistically significant indicators of a greater knowledge score. Contrary to other regional studies, including the one conducted among Qatari pharmacists, the results show that PharmD degree-holding pharmacists outscored their BSc counterparts [8]. This discrepancy may be attributed to differences in the integration of clinical pharmacogenomics within the specific pharmacy curricula of Jordanian versus Qatari universities. Moreover, the difference between our results and other studies may imply an important point: perhaps, mere training or availability of expertise may be irrelevant if this training is unstandardized, skill-based, and founded upon evidence-based clinical guideline evidence, an argument presented as a strong point against the effectiveness of training, which should be founded, instead, as stated, “upon the actionable, evidence-grade prescribing guidance offered by organizations, such as CPIC, rather than relying solely on pharmacogenomic biomarkers or cumulative allele counts” [2]. The inclusion of an “I don’t know” response option likely provided a better assessment, a conservative approach that approximated a truer measure by reducing potential guesses, lending further testament to this universal lack of PGx knowledge among pharmacists, strongly implying, as stated, a systemic problem, or, as indicated by regional literature reviews, gaps due to insufficient education or training [9].

One of the findings that emerged as a result of this study was the largely attitudinally positive approach Jordanian community pharmacists had towards the professional utility and clinical applications offered by PGx. It is noteworthy, however, that this attitude was coupled with a lack of knowledge, as established in the preceding section. The coupling of a positive attitude with a lack of knowledge is a characteristic that is apparent throughout the PGx literature around the world, as identified by a systematic review involving over 12,000 pharmacists and students [5]. This observation precisely matches the findings of the present study among Jordanian community pharmacists.

Such a scenario has been found to be prevalent among studies emerging from the Arab and Middle Eastern region. A study conducted among pharmacists and a study among doctors in Kuwait indicated a positive attitude toward PGx, even when their knowledge was low with minimal training related to PGx [6]. Likewise, a community pharmacists attitude study among UAE practitioners reported a favorable attitude alongside substantial gaps in their knowledge and attitude/practice, respectively [7]. The same studies emerging from this region emphasize how, even if the attitude is predominantly favorable, a number of barriers, including a lack of local guidelines, high costs, and a lack of reimbursement schemes, are influential, as identified as important components among our results as well [6,25].

The significance of having former PGx training as a predictive model for a greater positive attitude towards PGx (p = 0.04) was, arguably, the most interesting result emerging from this bivariate analysis. While this training failed to show a statistically significant influence on knowledge transfer, this was the first and, indeed, the only factor standing out as a strong predictor of a greater perceived positivity, an absolutely vital initial step towards potential implementation. As mentioned, this is a crucial point, well supported by the global literature, indicating a strongly positive correlation between any sort of PGx training, whether this be a matter of a curriculum component or a CPD session, and greater positivity and greater intention to make use of PGx services [26,27]. The evidence emerging relating to a greater positivity among those with former training may well be seen as a clear indication that education is the key to the initial, crucial winning over or buy-in process, well before any sort or level of substantive expertise can be achieved. While positivity, necessarily, does by no means imply a readiness level, this represents a natural transition point towards examining organizational, structural, and individual data informing a ready level.

Although pharmacists’ attitude towards PGx in our study was largely optimistic, the results regarding the attitude toward implementation readiness were a little complex and rather moderately optimistic. The overall scale score for implementation readiness, with a mean value of 3.34, showed a cautiously optimistic attitude, but this attitude did not demonstrate a high degree of readiness to implement their optimistic attitude toward the delivery of PGx care. This discrepancy is well-explained by implementation science frameworks, which posit that positive attitudes (i.e., motivation) are insufficient for practice change without a supportive organizational context and adequate resources [26]. When looking at the sub-constructs, a rather obvious picture emerges: the will exists, but the ways are inadequate.

The highest readiness score was attained by the Organizational Readiness scale, with a mean score of 3.39, indicating a moderately positive attitude toward organizations’ real commitment to implementing new services. It is well supported by the theory of the Organizational Readiness for Implementing Change, as readiness is described as a collective psychological state, implying that members feel both committed to and confident that they are able to make the change [20]. Nevertheless, case studies involving community-based PGx programs in community pharmacies often describe how this initial commitment may be threatened by difficulties between organizational workflows, documents, and professional interactions, all crucial organizational characteristics related to efficacy [18].

On the other hand, the lowest readiness ratings were perceived to be for both Structural & Practical Readiness and Leadership Support (M = 3.21 and 3.27, respectively). While these findings are based on high-impact proxy statements—a limitation that may not capture the full complexity of these constructs—they align with established implementation science frameworks which posit that positive attitudes (motivation) are insufficient for practice change without a supportive organizational context [26]. The argument given strongly supports this assertion by highlighting that the bivariate analysis established an insignificant relationship between total readiness for PGx implementation and any given demographic/practice characteristic, including the number of years practiced or an academic degree (p > 0.05 for all). The findings are consistent with the global trends, whereby PGx implementation among community pharmacists continues to face serious limitations in the following areas: lack of both the availability of PGx implementation time and availability of enough PGx implementation personnel, as well as a lack of both a PGx payment scheme or system and an integrated IT system that supports the PGx service [1,28].

The key point made by the ILS model, which assesses the extent to which leaders support evidence-based practice implementation, is the importance of active, knowledgeable leadership as an important driver of successful implementation [21]. The moderate score found in our study regarding leadership support suggests that, rather than obstructing, leadership may simply not yet be providing the strong, overt support, such as spending quality time or defining standard operating procedures, that distinguishes fully developed PGx programs from pilot programs [29].

Finally, the top-ranked individual challenge remained Privacy Concerns (M = 3.65), which is extremely consistent with the current body of literature. Patient and professional surveys repeatedly identify a concern for the privacy and confidentiality of genetic data as a determinant hindrance to the utilization of PGx resources [30]. The elevated concern, often described as “pharmacogenetic exceptionalism,” strongly attests to the paramount importance of a comprehensive framework regarding data governance, IT security, and established avenues of informed consent between pharmacists and their respective patients. The indication emerging among all mentioned findings, moderately-positive organizational readiness impaired by a lack of structural support and high concerns related to privacy, strongly suggests an identifiable scenario relative to PGx implementation within Jordan. The principal hindrances are, at this point, systemic rather than attitudinal.

As a reflection of their moderate level of readiness, participants were seen to rate the relative significance of barriers to providing PGx care as moderate, with a collective significance score of 3.45. Consistently with the regional body of research in Kuwait and Qatar [6,8], our findings highlight an alarmingly similar hierarchy of barriers. These include the lack of local guidelines, high implementation costs, and systemic hurdles to information access. This regional triangulation confirms that the difficulties faced by Jordanian pharmacists are far from exceptional; rather, they reflect a broader regional need to transcend the mere creation of awareness and concentrate on building standardized reimbursement and workflow solutions [31].

Such findings are also highly consistent with results seen among the region as a whole. A body of research among healthcare professionals in both Kuwait and Qatar has identified an alarmingly similar list of top-tier barriers, including a lack of training, a lack of a set structure or guideline, cost, and systemic barriers to access [6,8]. It is apparent that this regional triangulation has confirmed that the difficulties faced by pharmacists in Jordan are, far from being exceptional, something shared with other countries, and a number of systemic barriers that should be addressed if the goal of personalized medicine is to be achieved in the Middle East region are involved. It is obvious that the findings point to the need to transcend the mere creation of awareness and concentrate on building effective solutions, including reimbursement schemes, to achieve this goal.

One of the key aims of this research was to determine the independent predictors of PGx Knowledge, Attitudes, and Readiness. The multiple linear regression results represented a clear and conclusive response to this question: no demographic or practice-based characteristics within the variables tested were identified as significant independent predictors of PGx knowledge or readiness. While this suggests that the current readiness gaps are distributed broadly across the profession, the extremely low Adjusted R^2^ values indicate that these traditional variables explain a negligible percentage of the variance.

The only exception to this trend was identified in the bivariate analysis, which showed that prior PGx training was a statistically significant predictor of a more positive attitude (p = 0.04). However, this relationship did not remain significant in the multiple linear regression model (β = 0.09, p = 0.094), suggesting it may be influenced by other confounding variables. The implication is that this relationship was likely affected by other confounding variables. The overall implication, nonetheless, is that the hindrance was by no means reluctance, but rather a complete lack of training in PGx and a practice environment that is, as yet, not primed to execute the applications. When a person or a population, as a whole, has been exposed inadequately to a given subject matter, personal or professional characteristics, such as years of practice, are rendered irrelevant as a predictive marker for knowledge or readiness.

Despite being Jordan-specific, this finding, that personal and professional characteristics are not significant predictors of PGx knowledge and readiness, is consistent with the conclusions reached by the international literature as a whole. The results reached by systematic review around the world have repeatedly emphasized that PGx education experience is a central determinant both of knowledge and confidence [5]. Causal evidence, as gathered by interventional studies, confirms the effectiveness of PGx education programs, as they are able to significantly enhance both knowledge levels and confidence among pharmacists operating their professional activities [32]. It should thus be noted, rather than implying a lack of effectiveness, the lack of a strong signaling component in our results’ regression model essentially emphasizes that very few participants (17%) have ever received any sort of training at all, strongly implying the pressing necessity for a nationwide standardized training program as the initializing beginning for a professional, confident, and competent pharmaceutical care workforce to emerge and fully prepare for a world in which personalized medicine would be a reality.

The results and implications, including a landscape characterized by strong attitude patterns clouded by a lack of knowledge and readiness, pose clear guidance related to how a next-step approach may be taken to move the PGx service implementation process forwards in community pharmacies within Jordan. The findings strongly imply that this next step would require a simultaneous approach focusing on building attitude and enabler performance at all levels.

First, with regard to the universal knowledge gap, the development and scaling up of standardized, evidence-based education programs represents the highest priority. Based on the findings presented, even a small amount of training was a strong determinant for a greater degree of a positive attitude, making education a central concern for professional acceptance. The mentioned programs, whether incorporated into university courses or part of a professional development series, should be rooted in the evidence-based graded action items placed forth by CPIC, as mentioned by recent reviews, and should present a skills-based curriculum, accomplishing this aim via case studies, including competence development around result interpretation, as well as counseling and documentation [18].

Second, the organizational findings regarding readiness show a pressing necessity for a better-structured approach to implementation. The moderate ratings regarding organizational and leadership readiness make apparent the point that, although a degree of change willpower is present, a conducive structure is absent. The involvement and leadership support by pharmacy owners and managers should be a top concern, including providing a visible degree of protected time devoted to new services, development of goals, and promotion of PGx integration into the overall pharmacy processes [21]. The creation of Standard Operating Procedures (SOPs) related to the full-service chain, involving identification, consent, communication, and collaboration, represents a critical component toward diminishing the degree of resistance explored through structural findings related to readiness [28].

Finally, the critical barriers that arose as a result of this study are systemic/economic as well as others, as mentioned above. As a result, any strategy toward successful implementation should account for a viable route toward a reimbursement scheme involving pharmacists’ cognitive contributions to PGx. The inconclusivenessof a reimbursement scheme has been universally recognized as the major operating hindrance toward sustaining community pharmacy PGx initiatives around the world [33]. It is also important to actively direct efforts toward dispelling concern related to clients’ confidentiality [34]. As a result, rather than simple attitudinal readiness, this list provides a framework by which community pharmacy PGx can move forward as a capable, well-supported, and well-structured system suitable to the successful integration of PGx as a service component into the Jordanian community pharmacy arena.

The significant positive correlation found between Attitudes and Readiness (r = 0.42) provides a clear roadmap for policy. It suggests that while pharmacists have the will to implement services, this enthusiasm is currently decoupled from their know-how (as Knowledge did not correlate with Readiness). Therefore, our recommendation for a standardized national training program is not merely a general suggestion, but a direct response to this data: training must move beyond building positive sentiment and focus exclusively on clinical interpretation skills to bridge this specific knowledge gap. Furthermore, the high rating of Privacy Concerns (M = 3.65) necessitates the development of a specific PDPL-compliant data governance framework before any service launch.

There are a number of important strengths to this study that corroborate the findings as being valid. It appears this is the first national survey that explored PGx readiness, focusing exclusively on community pharmacists, that has taken place in Jordan. The stratified proportionate random sample approach taken in this study made the findings representative of the national community pharmacist population in Jordan. Additionally, the approach taken to create the instrument was robust; the approach to the assessment of knowledge was grounded in something meaningful, both clinically and evidence-based, utilizing the CPIC guidelines, and the readiness component was grounded in a theory, borrowing heavily from other successful scales, including the ORIC and the ILS [2,20,21].

However, the limitations of this study should also be recognized. Firstly, as a cross-sectional study, this research was able to establish links between findings, but any claim to a causal relationship could not be established. It could be suggested, for example, that a relationship exists between a lack of training and a negative attitude toward PGx, but this should not imply that a lack of training causes an adverse attitude toward PGx, as this could be the other way around or a number of other explanations could be applicable. Secondly, as this research relied on self-reporting, this could have resulted in a bias toward social desirability, whereby pharmacists could have reported their findings as they believed they should rather than as they actually are, as they wanted to appear as committed, knowledgeable, and professional as possible, even if they were really or perceived themselves as deficient in this regard. Thirdly, while we established content validity (S-CVI = 0.92), the test-retest reliability was performed on a small pilot sample, which may limit the assessment of long-term instrument stability. Moreover, the distribution of the survey via electronic platforms (email and WhatsApp) may have introduced selection and self-selection bias. Pharmacists with higher digital literacy or those who possess a baseline interest in innovative clinical services like PGx may have been more likely to participate than those who are less tech-savvy or less interested in the topic. This could potentially result in an overestimation of the positive attitudes and readiness scores reported in this study, as those with negative or indifferent views might have opted not to respond.

Finally, a limitation inherent to this study was the low values achieved by the regression equations for Adjusted R², reflecting that the measured demographic and practice variables did not explain the variance among the outcome values for the vast majority of the results. The likely explanation is that the actual values influencing PGx readiness, given the present state of the world, are unmeasured and individual, including, but not limited to, a pharmacist’s internal motivation, self-directed learning behavior patterns, such as reading professional publications or viewing web-based presentations, or their various experiences. The purpose of this research, as stated, was not necessarily to build a predictive model, but rather to offer a foundational, or so-called benchmark, model describing the present state-of-the-art present state of the profession.

While these models were non-significant, they indicate that traditional demographic and professional characteristics do not serve as primary drivers of PGx readiness in this population. This finding highlights a systemic gap that is universally distributed across the profession, though the low R^2^ suggests that unmeasured variables (e.g., personal motivation) may play a more significant role.

## Conclusion

In conclusion, this national survey of Jordanian community pharmacists provided a critical benchmark for the profession’s readiness to adopt pharmacogenomic services. The findings revealed a consistent pattern of low practical knowledge coexisting with generally positive attitudes. The readiness to implement PGx services was moderate overall, hindered by significant perceived barriers, particularly related to the high cost of tests, lack of reimbursement, and concerns regarding patient data privacy. The regression analyses were particularly revealing, demonstrating that the identified gaps in knowledge and readiness were universal across the profession and not concentrated in specific demographic or professional subgroups. The consistent absence of significant predictors reinforced the conclusion that a nationwide, standardized educational strategy is urgently required. To translate the existing positive sentiment into routine clinical practice, this educational push must be coupled with systemic enablers, including the development of reimbursement pathways, integrated clinical workflows, and explicit leadership support.

## Supporting information

S1 Table Raw data. (XLSX)

S1 File Questionnaire. (DOCX)
