# Supplementary material for: Pharmacogenomics knowledge and implementation readiness among community pharmacists in Jordan: A national cross-sectional study
Source: PLoS One. 2026 May 19;21(5):e0349439. doi: 10.1371/journal.pone.0349439 (PMC13186350; doi:10.1371/journal.pone.0349439)
Supplement: S1 File — (DOCX) [file pone.0349439.s002.docx]

# Questionnaire - Pharmacogenomics in Jordanian Community Pharmacy: A National Survey

### Section 0: Participant Information and Consent

Welcome,

You are invited to participate in a national research study. This study aims to understand the knowledge, attitudes, and readiness of community pharmacists in Jordan regarding a new area of personalized medicine called pharmacogenomics (PGx). Your professional insights are incredibly valuable for shaping the future of pharmacy practice in our country.

By clicking the "I agree to participate" button below, you are confirming that you have read the information above and voluntarily consent to participate in this research study.

- ( ) I agree to participate *(Proceed to Section A)*
- ( ) I do not agree to participate *(Survey ends)*

### Section A: Eligibility Screening

*This section ensures that your responses are included in the correct participant group.*

1. Are you currently a licensed pharmacist working in a community pharmacy in Jordan?

- ( ) Yes
- ( ) No *(Survey ends)*

2. What is your primary role in the pharmacy?

- ( ) Owner / Pharmacist-in-Charge / Manager
- ( ) Staff Pharmacist
- ( ) Intern / Trainee Pharmacist *(Survey ends)*

### Section B: Demographic and Practice Characteristics

1. What is your gender?

- ( ) Male
- ( ) Female

2. In which governorate is your primary pharmacy located?

- ( ) Central Region
- ( ) North Region
- ( ) South Region

3. Please select your highest pharmacy degree obtained:

- ( ) BSc in Pharmacy
- ( ) Doctor of Pharmacy (PharmD)
- ( ) Master's Degree (MSc) or higher

4. How many years have you been practicing as a licensed pharmacist?

- ( ) 0-5 years
- ( ) 6-10 years
- ( ) 11-20 years
- ( ) More than 20 years

5. Which of the following best describes your pharmacy?

- ( ) Independent
- ( ) Chain

6. Have you had any prior formal training (e.g., a workshop, a university course, a continuing education module) specifically on pharmacogenomics?

- ( ) Yes
- ( ) No

### Section C: Pharmacogenomic Knowledge Assessment

*Please choose the single best answer for each question. If you are unsure, please select "I don't know."*

1. Which of the following best describes the field of pharmacogenomics (PGx)?

- a) The study of how diseases affect a person's genetic makeup.
- b) The study of how a person's genes affect their response to medications.
- c) The study of how drugs are manufactured using genetic technology.
- d) I don't know.

2. A patient is a "CYP2D6 poor metabolizer." If this patient takes codeine (a prodrug activated by CYP2D6), what is the most likely outcome?

- a) Increased risk of toxicity from high morphine levels.
- b) Decreased or absent pain relief.
- c) No difference in effect compared to a normal metabolizer.
- d) I don't know.

3. Genetic testing for the *CYP2C19* gene is most critical for guiding the safe and effective use of which of the following medications?

- a) Metformin
- b) Clopidogrel
- c) Paracetamol
- d) I don't know.

4. A patient's PGx report for warfarin therapy would most importantly include information on which two genes?

- a) *CYP2D6* and *TPMT*
- b) *HLA-B* and *SLCO1B1*
- c) *CYP2C9* and *VKORC1*
- d) I don't know.

5. A patient is found to be a "CYP2C19 ultra-rapid metabolizer" and is prescribed the antidepressant citalopram. According to CPIC guidelines, what is the recommended action?

- a) Start with a lower-than-normal dose.
- b) Consider an alternative drug not metabolized by CYP2C19.
- c) Start with the standard dose.
- d) I don't know.

6. Pharmacogenetic testing is primarily used to:

- a) Diagnose genetic diseases like cystic fibrosis.
- b) Predict an individual's risk for future diseases.
- c) Predict how a patient will respond to a specific medication.
- d) I don't know.

7. A patient brings you a report showing they have the HLA-B*58:01 allele. Starting which of the following medications would carry a high risk of severe cutaneous adverse reactions (SCARs) for this patient?

- a) Simvastatin
- b) Allopurinol
- c) Amlodipine
- d) I don't know.

8. If a physician asks you for the most reliable, evidence-based resource for clinical guidance on how to act on a PGx result, which organization would you recommend?

- a) The World Health Organization (WHO)
- b) The Clinical Pharmacogenetics Implementation Consortium (CPIC)
- c) The drug's manufacturer website
- d) I don't know.

9. A patient who is a "TPMT poor metabolizer" is at a significantly increased risk of life-threatening myelosuppression if they take a standard dose of which medication?

- a) Warfarin
- b) Azathioprine
- c) Clopidogrel
- d) I don't know.

10. When counseling a patient about a PGx result, what is the most important concept to convey to avoid causing unnecessary alarm?

- a) That they have a "bad gene" which will affect many future medications.
- b) That a genetic variation is common and simply provides information to help the doctor choose the safest and most effective medicine for them personally.
- c) That they should avoid all medications metabolized by that specific enzyme for the rest of their life.
- d) I don't know.

### Section D: Attitudes and Perceptions

*Please indicate your level of agreement with the following statements on a scale from 1 (Strongly Disagree) to 5 (Strongly Agree).*

| Statement | Strongly Disagree (1) | Disagree (2) | Neutral (3) | Agree (4) | Strongly Agree (5) |
| --- | --- | --- | --- | --- | --- |
| 1. Integrating PGx services into my pharmacy practice would significantly improve patient safety. |  |  |  |  |  |
| 2. The clinical benefit of PGx testing is significant enough to justify the cost for many high-risk medications. |  |  |  |  |  |
| 3. It is my professional responsibility to be knowledgeable about pharmacogenomics. |  |  |  |  |  |
| 4. Physicians in Jordan would generally be receptive to PGx recommendations from community pharmacists. |  |  |  |  |  |
| 5. Counseling patients on their PGx results is a natural extension of the pharmacist's role in medication management. |  |  |  |  |  |
| 6. My patients would be interested in learning about how their genes affect their medications. |  |  |  |  |  |
| 7. Community pharmacists are well-positioned to lead the implementation of PGx services in primary care. |  |  |  |  |  |
| 8. Discussing genetic information with patients would enhance their trust in me as a healthcare professional. |  |  |  |  |  |
| 9. I believe that proactively recommending PGx testing for certain drugs would be a valuable service for my patients. |  |  |  |  |  |

### Section E: Barriers

1. Please rate how significant you believe each of the following would be as a barrier to offering PGx services in YOUR pharmacy.

| Barrier | Not a Barrier | A Minor Barrier | A Moderate Barrier | A Major Barrier | A Critical Barrier |
| --- | --- | --- | --- | --- | --- |
| 1. High out-of-pocket cost of the test for patients |  |  |  |  |  |
| 2. Lack of reimbursement for my time and expertise |  |  |  |  |  |
| 3. Lack of my own knowledge and training |  |  |  |  |  |
| 4. Lack of time in a busy pharmacy workflow |  |  |  |  |  |
| 5. Resistance or lack of collaboration from physicians |  |  |  |  |  |
| 6. Lack of clear clinical guidelines for our population |  |  |  |  |  |
| 7. Patient privacy and data security concerns (including risk of discrimination) |  |  |  |  |  |

### Section F: Implementation Readiness

Please indicate your level of agreement with the following statements on a scale from 1 (Strongly Disagree) to 5 (Strongly Agree).

| Statement | Strongly Disagree (1) | Disagree (2) | Neutral (3) | Agree (4) | Strongly Agree (5) |
| --- | --- | --- | --- | --- | --- |
| Organizational Readiness |  |  |  |  |  |
| **1.** My pharmacy's leadership and staff are generally committed to adopting new clinical services that improve patient care. |  |  |  |  |  |
| **2.** Overall, I believe my pharmacy has the capability (e.g., skills, resources) to successfully implement a new service like PGx. |  |  |  |  |  |
| Leadership Support |  |  |  |  |  |
| **3.** My immediate supervisor/owner would actively support me if I wanted to get trained and start offering PGx services. |  |  |  |  |  |
| Structural & Practical Readiness |  |  |  |  |  |
| **4.** My pharmacy has the physical space and workflow to accommodate private patient counseling for a service like PGx. |  |  |  |  |  |
| **5.** Our pharmacy's computer system is adequate for securely managing sensitive patient data like PGx results. |  |  |  |  |  |
| Privacy Concerns |  |  |  |  |  |
| **6.** I am concerned about the challenges of maintaining patient data privacy and complying with PDPL for a PGx service. |  |  |  |  |  |
| Personal Readiness and Intention |  |  |  |  |  |
| **7.** I would be willing to complete a formal certification program (e.g., 20 hours of training) to become qualified to offer PGx services. |  |  |  |  |  |
| **8.** I feel confident in my **current** ability to interpret a PGx report and effectively counsel a patient on the results. |  |  |  |  |  |
| **9.** I personally intend to seek out training or education in pharmacogenomics within the next 12 months. |  |  |  |  |  |
| **10.** It is likely that my pharmacy will actively explore offering PGx services within the next two years. |  |  |  |  |  |

Thank You!

Thank you for taking the time to complete this important survey. Your contribution is invaluable to advancing the pharmacy profession in Jordan. You may now close this window.
